# Wogonin induces ferroptosis in pancreatic cancer cells by inhibiting the Nrf2/GPX4 axis

**DOI:** 10.3389/fphar.2023.1129662

**Published:** 2023-02-22

**Authors:** Xing Liu, Xinhui Peng, Shuai Cen, Cuiting Yang, Zhijie Ma, Xinyuan Shi

**Affiliations:** ^1^ School of Chinese Materia Medica, Beijing University of Chinese Medicine, Beijing, China; ^2^ Department of Pharmacy, Beijing Friendship Hospital, Capital Medical University, Beijing, China; ^3^ School of Life Science, Beijing University of Chinese Medicine, Beijing, China

**Keywords:** pancreatic cancer, wogonin, ferroptosis, Nrf2, lipid peroxidation

## Abstract

Pancreatic cancer is a common gastrointestinal tract malignancy. Currently, the therapeutic strategies for pancreatic cancers include surgery, radiotherapy, and chemotherapy; however, the surgical procedure is invasive, and the overall curative outcomes are poor. Furthermore, pancreatic cancers are usually asymptomatic during early stages and have a high degree of malignancy, along with a high rate of recurrence and metastasis, thereby increasing the risk of mortality. Studies have shown that ferroptosis regulates cell proliferation and tumour growth and reduces drug resistance. Hence, ferroptosis could play a role in preventing and treating cancers. Wogonin is a flavonoid with anticancer activity against various cancers, including pancreatic cancer. It is extracted from the root of *Scutellaria baicalensis Georgi*. In this study, we show that wogonin inhibits the survival and proliferation of human pancreatic cancer cell lines and induces cell death. We performed RNA-sequencing and analysed the differentially expressed gene and potential molecular mechanism to determine if wogonin reduced cell survival *via* ferroptosis. Our results showed that wogonin upregulates the levels of Fe^2+^, lipid peroxidation and superoxide and decreases the protein expression levels of ferroptosis suppressor genes, and downregulates level of glutathione in pancreatic cancer cells. In addition, ferroptosis inhibitors rescue the ferroptosis-related events induced by wogonin, thereby confirming the role of ferroptosis. A significant increase in ferroptosis-related events was observed after treatment with both wogonin and ferroptosis inducer. These results show that wogonin could significantly reduces pancreatic cancer cell proliferation and induce ferroptosis *via* the Nrf2/GPX4 axis. Therefore, wogonin could be potentially used for treating patients with pancreatic cancer.

## Introduction

A significant improvement in the patient survival rate is observed due to the rapid advancement in modern medical techniques and cancer therapeutics. The diagnosis of patients at an early stage is difficult; hence, the 5-year survival rate of patients with pancreatic cancer is extremely low, and mortality is high (Ansari et al., 2016). Based on a survey conducted by the American Cancer Society in 2021, pancreatic cancer ranks seventh in cancer-related mortality (Sung et al., 2021). A study has shown that pancreatic cancer will be the second leading cause of cancer-related mortalities in the United States by 2030 (Rahib et al., 2014). Furthermore, pancreatic cancer is anticipated to surpass breast cancer as the third leading cause of cancer-related death in the European Union by 2021 (Carioli et al., 2021). A few challenges associated with pancreatic cancer treatment include a limited number of chemotherapy drugs available and drug resistance, especially resistance to gemcitabine (Qin et al., 2020). Therefore, developing and designing therapeutic strategies with good efficacy and low drug resistance for treating patients with pancreatic cancer is urgently required.

Recent studies have shown that ferroptosis is a form of cell death and differs from necrosis, apoptosis, and pyroptosis (Koren and Fuchs, 2021). The mechanism of ferroptosis regulation consists of four aspects: 1) Iron metabolism; 2) The GSH system; 3) The BH4/CoQ_10_ system; 4) Transcriptional regulation (Chen et al., 2022). Therefore, an imbalance in four regulatory pathways could significantly reduce the activity of GPX4 and increase the level of intracellular lipid ROS, which reduces the antioxidant activity of cells. Hence, the accumulation of additional lipid ROS could trigger ferroptosis. With the deepening of research, it has been found that ferroptosis not only effectively kills cancer cells, but also plays an important role in inhibiting tumor cell migration (Mou et al., 2019), reversing tumor drug resistance (Viswanathan et al., 2017), and improving tumor immune response (Bi et al., 2022). In addition, ferroptosis-based nanoparticle inducers are more effective and have fewer side effects than traditional chemotherapy drugs (Zhang et al., 2020). Designing combination therapies based on ferroptosis processes in combination with nanomedicine is also an attractive line of research. Natural products, such as flavonoids, quinones, alkaloids, terpenoids, saponins, polysaccharides, polyphenols, and lignans, can increase intracellular reactive oxygen species and disrupt redox homeostasis (Wu et al., 2022). However, the pharmacological mechanism of these substances in ferroptosis still needs further study.

**FIGURE 1:**
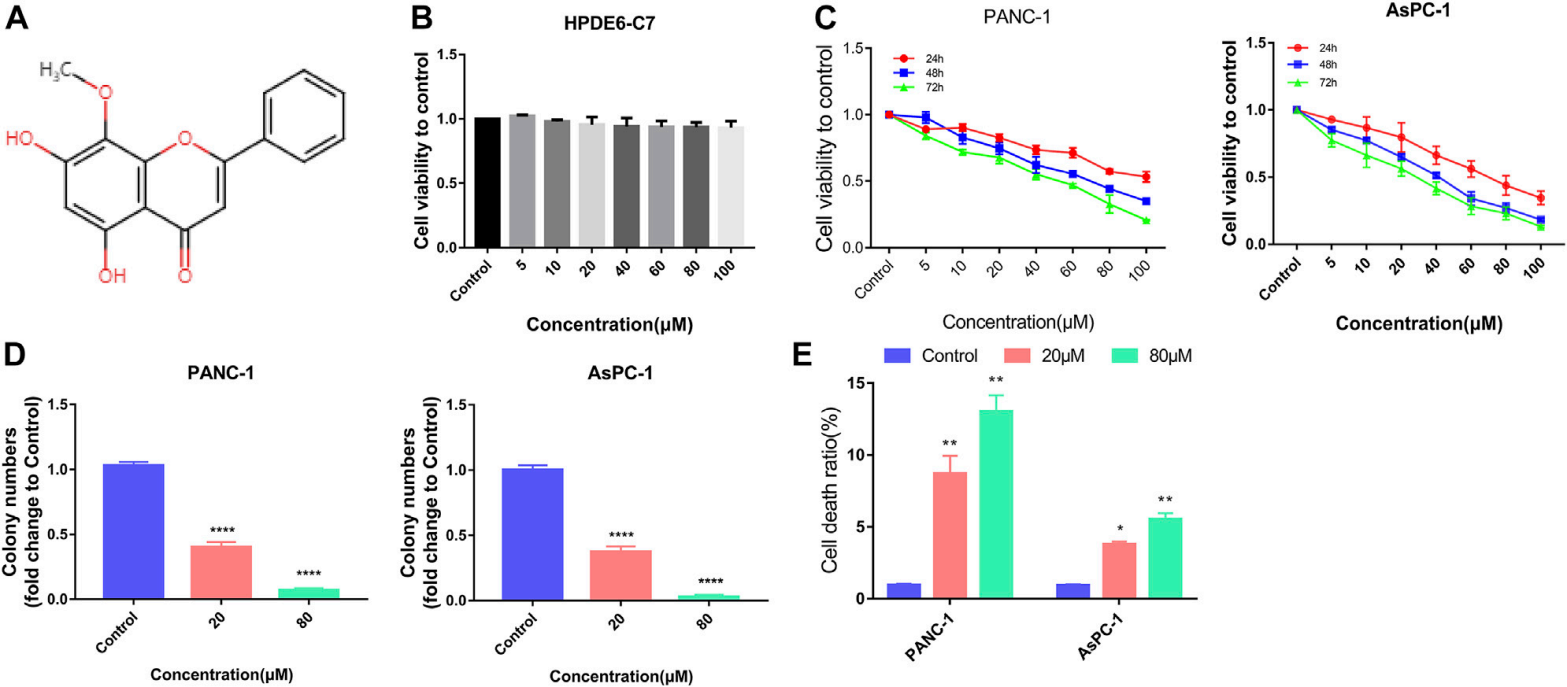
Wogonin inhibited viability and induced death in pancreatic cancer cells. **(A)** Chemical structure of wogonin. **(B)** CCK8 assay proved that treatment with wogonin for 48 h had little toxic effect on human pancreatic duct epithelial cell line (HPDE6-C7). **(C)** CCK8 assay showed that wogonin inhibited the viabilities of PANC-1 and AsPC-1 pancreatic cancer cells in a dosage- and time-dependent manner. **(D)** Colony formation assay proved that 20 μM wogonin could obviously inhibit PANC-1 and AsPC-1 pancreatic cancer cells to form colonies, which became more apparent when wogonin dosage was increased to 80 μM. **(E)** LDH release assay demonstrated that wogonin triggered dosage-dependently pancreatic cancer cell death. *: *p* < 0.05 versus control group, **: *p* < 0.01 versus control group. ****: *p* < 0.0001 versus control group.

In China, for over 1,000 years, *Scutellaria baicalensis Georgi* has been used as a traditional Chinese medicine for treating cancers, diabetes, *etc.* (Zhou et al., 2021). One of the flavonoids extracted from *S. baicalensis Georgi* is wogonin (Figure 1A) (Liu et al., 2022), which possesses several properties like neuroprotection (Smith et al., 2020), anticancer, anti-inflammatory, and antiviral (Cho and Lee, 2004). Several studies have reported that wogonin exhibits significant anticancer effects in multiple diseases like breast cancer (Yang et al., 2020), colorectal cancer (Feng et al., 2018), cervical cancer (Kim et al., 2013), leukaemia (Cao et al., 2020), gastric cancer (Hong et al., 2018), liver cancer (Hong et al., 2020), lung cancer (Wang et al., 2021), glioblastoma (Lee et al., 2012), and osteosarcoma (Huynh et al., 2017). Wogonin promotes pancreatic cancer cell death by increasing ROS levels (Li et al., 2016). Nuclear factor E2-related factor 2 (Nrf2) is a key transcription factor in the regulation of antioxidant stress response. While protecting normal cells from DNA damage induced by reactive oxygen species, malignant tumor cells are also protected. Nrf2 regulates ferroptosis by targeting many genes involved in iron/metal metabolism, such as ferritin light and heavy chains (FTL/FTH1), SLC40A1, heme oxygenase-1 (HO-1), biliverdin reductase A and B (BLVRA/B), ferrochelatase (FECH), ATP-binding cassette subfamily B member 6 (ABCB6), and SLC48A1 (Shi et al., 2021). Therefore, blocking Nrf2 expression during ferroptosis targeting therapy is critical. In addition, Nrf2 expression level was high in more than 93% of pancreatic adenocarcinomas (Dai et al., 2021). Nrf2 overexpression is thought to be the result of the almost universal oncogenic *KRAS* gene mutation and downstream activation of the MAPK pathway as well as high levels of c-Myc (Cykowiak and Krajka-Kuźniak, 2021). Notably, high nuclear expression of Nrf2 is associated with reduced survival in patients with pancreatic cancer. Targeting Nrf2 may be an effective therapeutic strategy in pancreatic cancer. However, the involvement of ferroptosis and Nrf2 in the wogonin-mediated death of pancreatic cancer cells is still unclear. Therefore, our study aims to investigate whether wogonin could induce ferroptosis and its underlying mechanism using human pancreatic cancer cell lines and xenograft mice models of pancreatic cancers.

## Materials and methods

### Reagents

Dulbecco’s Modified Eagle’s Medium (DMEM, Biological Industries, Beit Haemek, Israel), foetal bovine serum (FBS, Biological Industries, Israel), Roswell Park Memorial Institute (RPMI) 1,640 medium (Biological Industries, Israel), Lipo6000™ Transfection Reagent (Beyotime Biotechnology, China), Phosphate-buffered saline (PBS, Beyotime Biotechnology, China), Modified Giemsa Staining Solution (Beyotime, C0131), Caspase Inhibitor Z-VAD-FMK (Beyotime Biotechnology, China), Lactate Dehydrogenase Cytotoxicity Assay Kit (Beyotime Biotechnology, China), ROS Assay Kit (Beyotime Biotechnology, China) and Superoxide Assay Kit (Beyotime Biotechnology, China), GSH assay kit (Nanjing Jiancheng Bioengineering Institute, China), Malondialdehyde assay kit (Nanjing Jiancheng Bioengineering Institute, China), and tissue iron assay kit (Nanjing Jiancheng Bioengineering Institute, China), Cell counting kit-8 (CCK-8, CK04), Liperfluo (L248), and FerroOrange (F374) were obtained from Dojindo Laboratories (Shanghai, China). Wogonin (B20489) and ferroptosis inducers like erastin (S80804), L-buthionine-sulfoximine (BSO, S51087), FIN56 (S81914), FAC (S24249), and ferroptosis inhibitors like ferrostatin-1 (Fer-1, S81461), and deferoxamine (DFO, S61301) were procured from Shanghai Yuanye Bio-Technology Co. Ltd. (Shanghai, China). Primary antibodies like β-actin (20536-1-AP), HO-1 (10701-1-AP), solute carrier family 7 member 11 (SLC7A11, 26864-1-AP), GPX4 (67763-1-Ig), and Nrf2 (16396-1-AP) were procured from Proteintech (Wuhan, China). Highly analytical chemical reagents were used in the present research.

### Cell culture

PANC-1 and AsPC-1 cells were used due to their clear genetic background and availability. Human pancreatic ductal adenocarcinoma (PAAD) cells: AsPC-1, PANC-1, and HPDE6-C7 (human normal pancreatic epithelial cells) were provided by BeNa Culture Collection (Xinyang, China). HPDE6-C7 and AsPC-1 cells were cultured and maintained in RPMI 1640 medium. DMEM was used for culturing PANC-1 cells.

### Cell viability, colony formation, and cell death assays

Cell counting kit-8 (CCK-8) was used to measure the cell viability, which was expressed as a ratio of the absorbance measured at 450 nm in treated and control cells.

For conducting colony formation assay: pancreatic cancer cells were seeded into a 6-well plate at a density of 2,000 cells/well for 24 h and treated with 20 μM and 80 μM wogonin or 0.1% dimethyl sulfoxide (in culture medium) as a control for 5 days. The cells were fixed in methanol for 10 min and stained using crystal violet (200 μg/mL) for 20 min. Colonies with >50 cells were counted using a microscope.

To determine cell death, the LDH activity released from damaged cells were measured at different time points using the LDH cytotoxicity detection kit as per the manufacturer’s protocol.

### RNA sequencing after treating PANC-1 with wogonin

RNA sequencing was performed by means of Novogene RNA sequencing. The cells were treated with 80 μM wogonin and without wogonin for 24 h. Next, the total RNA was isolated using the TRIzol reagent. 2 μg total RNA/sample was used for sample preparation for RNA sequencing. Briefly, poly-T oligonucleotide-linked magnetic beads were used for purifying mRNA from the total RNA. The first complementary DNA (cDNA) strand was synthesised using random hexamers plus M-MuLV reverse transcriptase. DNA polymerase I, along with RNase H, was used for the second cDNA strand synthesis. The amplified polymerase chain reaction (PCR) product was purified using the AMPure XP system. The “DESeq2” R package (1.20.0) was used for analysing differentially expressed genes between the two conditions/groups (two biological replicates were used/condition). The “clusterProfiler” R package was used to perform Gene ontology (GO) on differentially expressed genes (DEGs) and Kyoto Encyclopedia of Genes and Genomes (KEGG) pathway enrichment for analysing the pathways significantly enriched by DEGs (|Log2 FC| >1). The gene length deviation was corrected. The expression of transcripts with adjusted *p* < 0.05 was considered statistically significant.

### ROS and lipid peroxidation quantification assay

For ROS detection, PAAD cells were treated with 10 μM H2DCFDA at 37°C for 30 min. For lipid peroxidation, the cells were stained with 1 μM Liperfluo at 37°C for 30 min, and the fluorescence intensity were measured. Subsequently, the cells were washed with Hanks’ Balanced Salt Solution (HBSS) twice and analysed using flow cytometry.

### Superoxide assay

The superoxide levels in the cells were measured using a superoxide assay kit as indicated by the manufacturer. Briefly, 8 × 10^3^ PAAD cells/well were cultured in 96-well plates and were treated as indicated. Subsequently, the superoxide detection reagent (200 μL/well) was added to the cells and incubated at 37°C for 3 min. The absorbance of cells was measured at 450 nm for the superoxide assay.

### Iron assay

To determine if wogonin regulates ferrous (Fe^2+^) ion production, we used FerroOrange (an intracellular Fe^2+^ ion probe) assay to measure the iron levels in cells. Briefly, 1 × 10^4^ PAAD cells were harvested and washed with HBSS thrice. Next, the cells were incubated with 1 μM FerroOrange (Ex: 543 nm and Em: 580 nm) in serum-free medium at 37°C for 30 min in an incubator with 5% CO_2_. The fluorescence microplate reader was used to measure the fluorescence intensity.

### GSH assay

The total glutathione levels were measured using a GSH Assay Kit. In addition, the levels were normalised by cell number based on the manufacturer’s instructions.

### Reverse transcription-quantitative polymerase chain reaction (RT-qPCR)

Total RNA was isolated using the TRIzol reagent (Sigma-Aldrich, St. Louis, MO, United States) following the manufacturer’s protocol and reverse transcribed using a Prime Script RT-PCR Kit (ChamQ SYBR Color qPCR Master Mix, Vazyme, Nanjing, China) for cDNA synthesis. The RT-qPCR was performed using StepOnePlus™ real-time system (Applied Biosystems™, CA, United States) and SYBR mix. The cyclic conditions for PCR amplification were based on the manufacturer’s instructions.

The primer sequence is as follows:

*NFE2L2* forward primer (FP): 5′-TAG​AGT​CAG​CAA​CGT​GGA​AG-3′

Reverse primer (RP): 5′-TAT​CGA​GGC​TGT​GTC​GAC​TG-3′

*SLC7A11* FP: 5′-GCT​GAC​ACT​CGT​GCT​ATT-3′

RP: 5′-ATT​CTG​GAG​GTC​TTT​GGT-3′ and

*HO-1* FP: 5′-TAG​AGT​CAG​CAA​CGT​GGA​AG-3′

RP: 5′-TAG​AGT​CAG​CAA​CGT​GGA​AG-3′.

### Interference and overexpression of genes

Small interfering RNA (siRNA) was transfected in cells using Lipo8000™ Transfection Reagent (Beyotime) as indicated by the manufacturer; however, the protocol was modified as required. The siRNAs were purchased from Ribobio (Guangzhou, China). *NFE2L2* siRNAs sequence was 5′-GAG​AAA​GAA​TTG​CCT​GTA​A-3′, and scrambled siRNA sequence (used as negative control) was 5′-GGC​UCU​AGA​AAA​GCC​UAU​GCT​T-3′. A lentivirus vector was used for overexpressing *NFE2L2* (*NFE2L2*-OE) in cells, and a negative vector (*NFE2L2* -NC) was used as a control. Both vectors were synthesised by Miaolinbio (Wuhan, China). Vector transduction at a multiplicity of infection (MOI) of 1, 10, and 30 was performed, and the optimal MOI was identified as 30. For *NFE2L2* overexpression, the pancreatic cancer cells were seeded and cultured in 6-well plates until the cells were 30%–50% confluent. The cells were transfected with lentiviral vectors at an MOI of 30, and the culture medium was replaced after 24 h. The transfection was performed in triplicates. The transfection efficiency was determined using RT-qPCR and western blotting (WB). The pancreatic cancer cells with stable *NFE2L2*-OE were selected by treating the cells with puromycin (2 μg/mL) for 24 h.

### *In vivo* pancreatic cancer mice model

Female BALB/c nude mice (5 weeks old) were procured from Hangzhou Ziyuan Laboratory Animal Technology Co., Ltd (Zhejiang, China) and given 5 days to acclimate to their surroundings. PANC-1 cells (1 × 10^7^) in 100 μL PBS at the logarithmic growth phase were administered to mice subcutaneously in the left flank. The mice were treated with indicated treatments after nearly 10 days when the tumour size was approximately 1,000 mm^3^. In the control group, mice (*n* = 5) received intraperitoneal injections of the vehicle. In the treatment group, the mice (*n* = 5) were administered 50 μL of 60 mg/kg body weight of wogonin once a day for 12 days. A slide calliper size was used to measure the tumour size. The equation for calculating tumour volume is as follows: tumour volume = AB^2^/2, wherein A is the length, and B is the width of the tumour. The mice were sacrificed the next day after the treatment procedure was complete by cervical dislocation. The tumour tissues were harvested and snap-frozen using liquid nitrogen for subsequent analyses. All the procedures involving animals were reviewed and approved by the Animal Care and Use Committee of the Beijing University of Chinese Medicine (the ethical approval number: BUCM-2021101301-3011).

### Western blot analysis

Western blot was performed as per standard protocol. Briefly, the protein was extracted from the tumour tissue-cell lysate. 30 μg proteins were separated by SDS-PAGE, and the proteins were transferred onto polyvinylidene fluoride membranes. The following primary antibodies were used: Nrf2 at 1:1,000 dilution (ab137550, Abcam, United Kingdom), SLC7A11 at 1:1,000 dilution (ab37185, Abcam, United Kingdom), GPX4 at 1:1,000 dilution (ab125066, Abcam, United Kingdom), HO-1 and β-Actin at 1:1,000 dilution (70,081 and 4970S, respectively from Cell Signalling Tech, United States). The protein bands were detected using chemiluminescence (Millipore, MA, United States) and exposed to X-ray film (RX-U; Fujifilm, China).

### Histological examination

First, 4 mm paraffin-embedded sections of the mouse tissue were made. The sections were stained with haematoxylin and eosin for histological evaluation of the ischemia-reperfusion injury. The protocol for histological evaluation was described in the Supplementary Materials.

### Statistical analysis

GraphPad Prism 7.0 software (GraphPad, United States) was used to perform data analysis. Test like one-way analysis of variance (ANOVA) was used to perform analysis of the experimental data. All data were represented as the mean ± SD (standard deviation). *p* < 0.05 was considered statistically significant.

## Results

### Wogonin reduces cell viability and induces pancreatic cancer cell death

The cytotoxic and inhibitory effects of wogonin on HPDE6-C7, PANC-1 and AsPC-1 cell proliferation were determined. The cells were treated with 0, 5, 10, 20, 40, 60, 80, and 100 μM concertation of wogonin at different time points. The cell viability was measured using CCK-8 assays, and the results revealed no significant differences in HPDE6-C7 cell viability on treatment with different doses of wogonin after 48 h (Figure 1B). However, a significant decrease in the viability and proliferation of PANC-1 and AsPC-1 cells was observed on treatment with wogonin in incubation time- and concentration-dependent manner (Figure 1C). Since wogonin treatment reduces PANC-1 and AsPC-1 cell viability, the IC_50_ values of wogonin were calculated. The IC_50_ values of wogonin were 103.6 μM for PANC-1 cells and 84.71 μM for AsPC-1 cells. Therefore, 80 μM as the IC_50_ value of wogonin was used for treating PANC-1 and AsPC-1 cells for subsequent experiments. To determine the effect of wogonin on cell proliferation, we performed the colony-formation assay. The results revealed a significant decrease in the colony-forming ability of PANC-1 and AsPC-1 cells on treatment with wogonin (Figure 1D). Furthermore, the LDH activity release assay showed that wogonin induced PANC-1 and AsPC-1 cell death in a concentration-dependent manner at 24 h (Figure 1E). In summary, wogonin showed limited cytotoxic effects on HPDE6-C7 but could significantly reduce PANC-1 and AsPC-1 cell viability and induced cell death.

### Involvement of ferroptosis in wogonin-induced pancreatic cancer cell death

**FIGURE 2:**
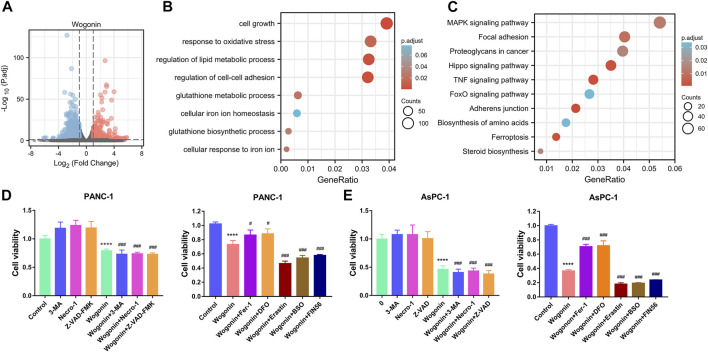
Screening of the death mode of pancreatic cancer cells induced by wogonin. **(A)** The genes regulated by wogonin in PANC-1 cells. **(B)** Enrichment analysis of GO in wogonin group compared with control group assayed by RNA-sequence in PANC-1 cells. **(C)** Enrichment analysis of KEGG signaling pathway in wogonin group compared with control group assayed by RNA-sequence in PANC-1 cells. **(D)** Effects of three inhibitors on the death of pancreatic cancer cells induced by wogonin. **(E)** Effects of ferroptosis inhibitors on the death of pancreatic cancer cells induced by wogonin. ****p < 0.0001 versus control group. #*p* < 0.05 versus wogonin-alone group; ###*p* < 0.001 versus wogonin-alone group.

To explore the underlying mechanisms of wogonin-induced pancreatic cancer cell death, RNA-sequencing was performed on PANC-1 cells treated with 80 μM of wogonin. The results revealed that 3667 DEGs were identified, among which 1,589 DEGs were upregulated and 2078 DEGs were downregulated (Figure 2A). Furthermore, the DEGs were subjected to the GO and KEGG pathway enrichment analysis. The results revealed that the DEGs significantly enriched the ferroptosis pathway (Figures 2B, C). Therefore, these results imply that ferroptosis could be a key regulator of pancreatic cancer cell death due to wogonin.

To further determine the specific mode of cell death induced by wogonin treatment, we treated PANC-1 and AsPC-1 cells with wogonin in the presence or absence of apoptosis, necrosis, and autophagy inhibitors. The cells were treated with Z-VAD-FMK, necroptosis inhibitors like necrostatin-1 or 3-Methyladenine (autophagy inhibitor). The results revealed that the treatment with these inhibitors could not protect against the death of pancreatic cancer cells induced by wogonin (Figure 2D), thereby indicating that wogonin used other mechanisms to induce tumour cell death. Further, PANC-1 and AsPC-1 cells were treated with ferroptosis inhibitor agents like DFO or Fer-1 and ferroptosis-inducing agents like erastin, BSO, or FIN56. The results revealed that treatment with 200 µM DFO or 1 μM Fer-1 almost completely attenuated wogonin-induced cancer cell death and 10 µM erastin, 200 μM BSO, or 150 μM FIN56 accelerate to wogonin-induced PANC-1 and AsPC-1 cell death (Figure 2E). Together, these results indicate the involvement of ferroptosis in the wogonin-induced pancreatic cancer cell death.

### Iron-regulated wogonin-induced pancreatic cancer cell death

**FIGURE 3:**
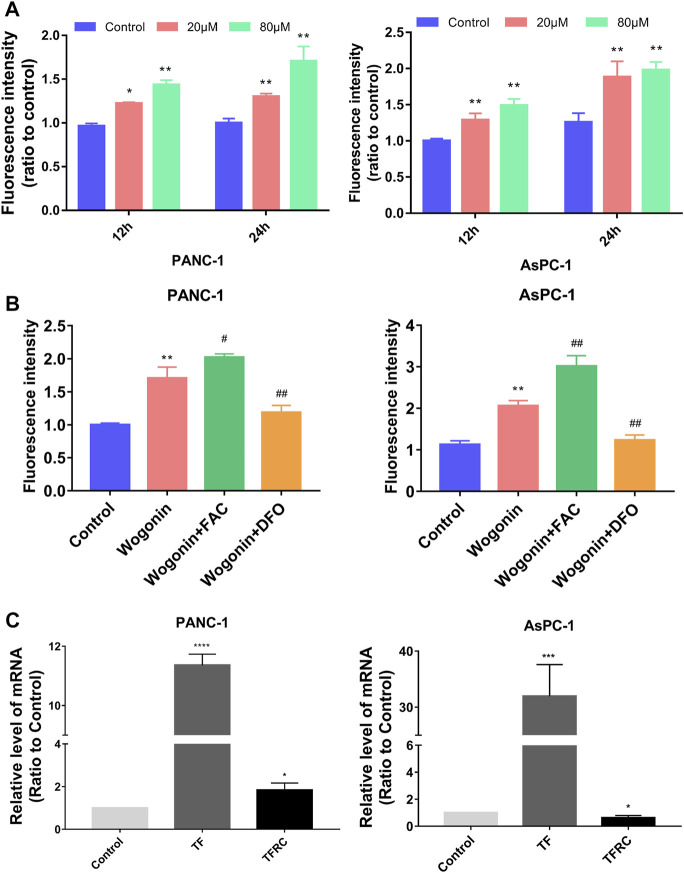
Fe^2+^ regulated wogonin-induced pancreatic cancer cell death. **(A)** Intracellular Fe^2+^ levels in pancreatic cancer cells. **(B)** Fe^2+^ assay revealed that wogonin-induced increase of ferrous iron was inhibited by deferoxamine (DFO), but reinforced by ferric ammonium citrate (FAC). **(C)** RT-qPCR analysis revealed that wogonin triggered time-dependent upregulation of transferrin receptor (TFR), transferrin (TF). *p < 0.05 versus control group; **: *p* < 0.01 versus control group; ***: *p* < 0.001 versus control group; ****: *p* < 0.0001 versus control group; #*p* < 0.05 versus wogonin-alone group; ###*p* < 0.001 versus wogonin-alone group.

We measured the levels of Fe^2+^ ions in cells to determine if wogonin could induce ferroptosis in the pancreatic cancer cells compared to the control cells. An increase in Fe^2+^ ion levels in both AsPC-1 and PANC-1 cells was observed on treatment with 20 μM wogonin for 12 h. Moreover, Fe^2+^ ions levels increased further in PANC-1 and AsPC-1 cells on treatment with 80 μM wogonin concentration or increased in the incubation time to 24 h. These results indicate that wogonin increased Fe^2+^ ion concentration in cells in a time as well as concentration-dependent manner (Figure 3A).

To determine the significance of the increase in Fe^2+^ ion concentration, the cells were first treated with 200 μM DFO for 1 h, followed by treatment with 80 μM wogonin for 24 h. The results revealed that a DFO significantly inhibited Fe^2+^ levels in cells, which were high due to wogonin treatment. However, pre-treatment with 200 μM FAC for 1 h significantly enhanced the toxicity of wogonin on pancreatic cancer cells (Figure 3B). These results indicate that wogonin-induced pancreatic cancer cell death by increasing the levels of Fe^2+^ ions in cells. RT-qPCR was used to identify the factors regulating the increase in the levels of Fe^2+^ ions in AsPC-1 and PANC-1 cells upon wogonin treatment. The results showed an increase in the expression of TF (binds to iron) and TFRC (transports the TF-iron complex into cells) in AsPC-1 and PANC-1 cells on treatment with 80 μM wogonin in a time-dependent manner (Figure 3C). This indicates that wogonin induces the accumulation of Fe^2+^ ions in pancreatic cancer cells by increasing the influx of iron into cells.

### Wogonin induces pancreatic cancer cell death by increasing lipid peroxidation and reducing GSH levels

**FIGURE 4:**
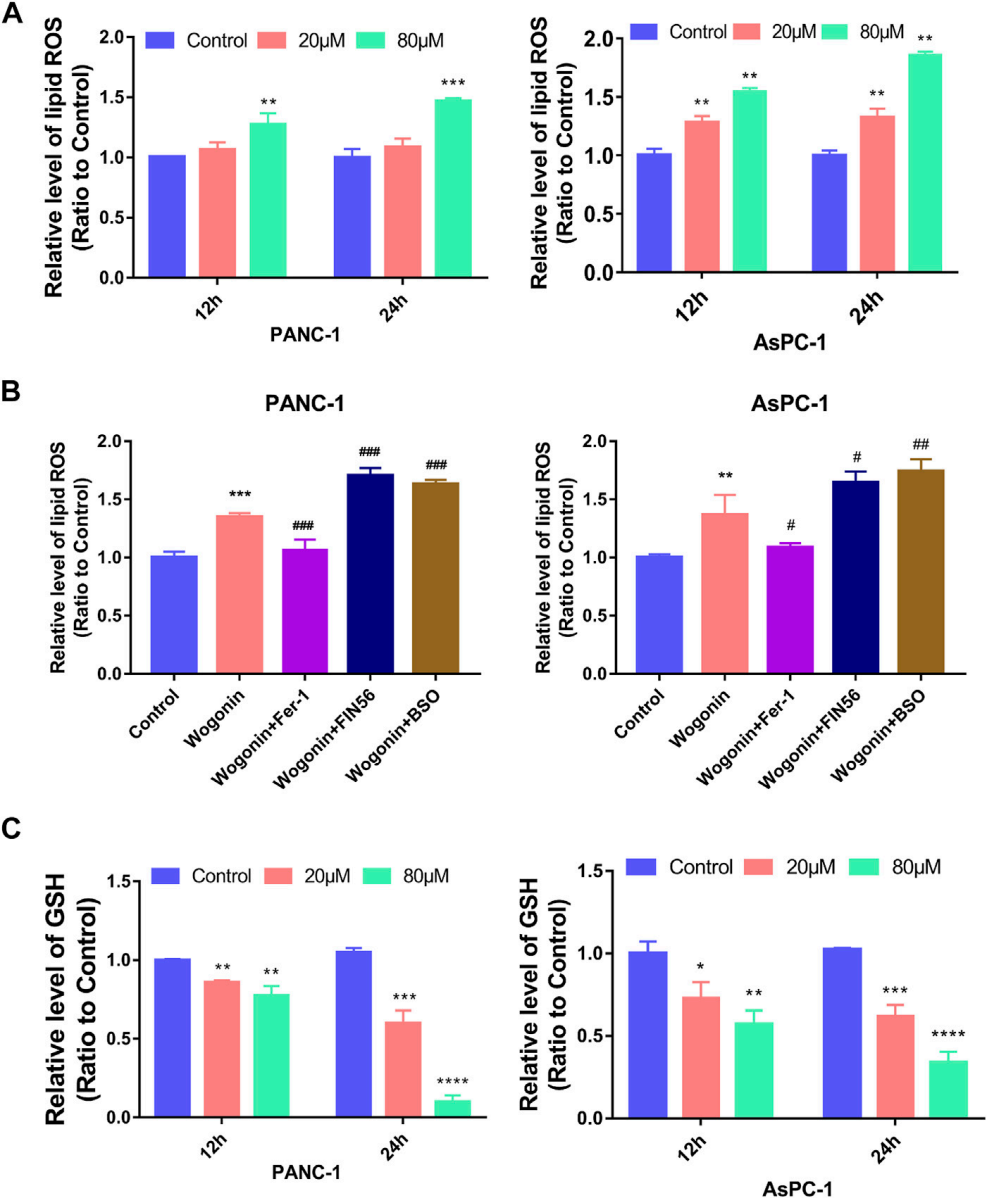
Wogonin induced lipid peroxidation and GSH changes in pancreatic cancer cells. **(A)** Wogonin induced accumulation of lipid peroxidation in pancreatic cancer cells in a dosage- and time-dependent manner. **(B)** The upregulation of lipid peroxidation caused by wogonin was prevented by pretreatment with Fer-1, but was promoted by BSO or FIN56. **(C)** Wogonin reduces the level of GSH in pancreatic cancer cells in a dosage- and time-dependent manner. *p < 0.05 versus control group; **: *p* < 0.01 versus control group; ***: *p* < 0.001 versus control group; ****: *p* < 0.0001 versus control group; #*p* < 0.05 versus wogonin-alone group; ###*p* < 0.001 versus wogonin-alone group.

Lipid peroxidation is an important aspect of ferroptosis; hence, we examined the changes in lipid peroxidation in cells on treatment with wogonin. The results showed a significant increase in lipid peroxidation in cells on treatment with 20 μM wogonin for 12 h. The lipid peroxidation increased further when the concertation of wogonin was increased to 80 μM or the incubation time was increased to 24 h (Figure 4A). This indicates dose and time-dependent increase in lipid peroxidation in cells on treatment with wogonin.

To investigate if lipid peroxidation is involved in wogonin-induced pancreatic cancer cell death, the cells were first treated with 1 μM Fer-1 for 1 h, followed by treatment with 80 μM wogonin for 24 h. The results revealed that a significant reduction in lipid peroxidation due to wogonin treatment was observed when the cells were pretreated with Fer-1 for 1 h. Similarly, pre-treatment with 200 μM BSO or 150 μM FIN56 for 1 h could promote the lethal effect of wogonin treatment on pancreatic cancer cells (Figure 4B). Together, our results indicate the involvement of lipid peroxidation in pancreatic cancer cell death following wogonin treatment.

GSH is a GPX4-reducing agent that catalyses lipid peroxides reduction to its corresponding alcohols, thereby protecting cells from damage due to lipid peroxidation and inhibiting ferroptosis in cells. Hence, we measured GSH levels in wogonin-treated cells. The results revealed a significant decrease in GSH levels in cells treated with 20 μM wogonin for 12 h, which was further aggravated by an increase in incubation time to 24 h or wogonin dose to 80 μM (Figure 4C). This indicates that wogonin reduces GSH levels in cells in a time- and concentration-dependent manner.

### Wogonin treatment increases the production of superoxide and ROS

**FIGURE 5:**
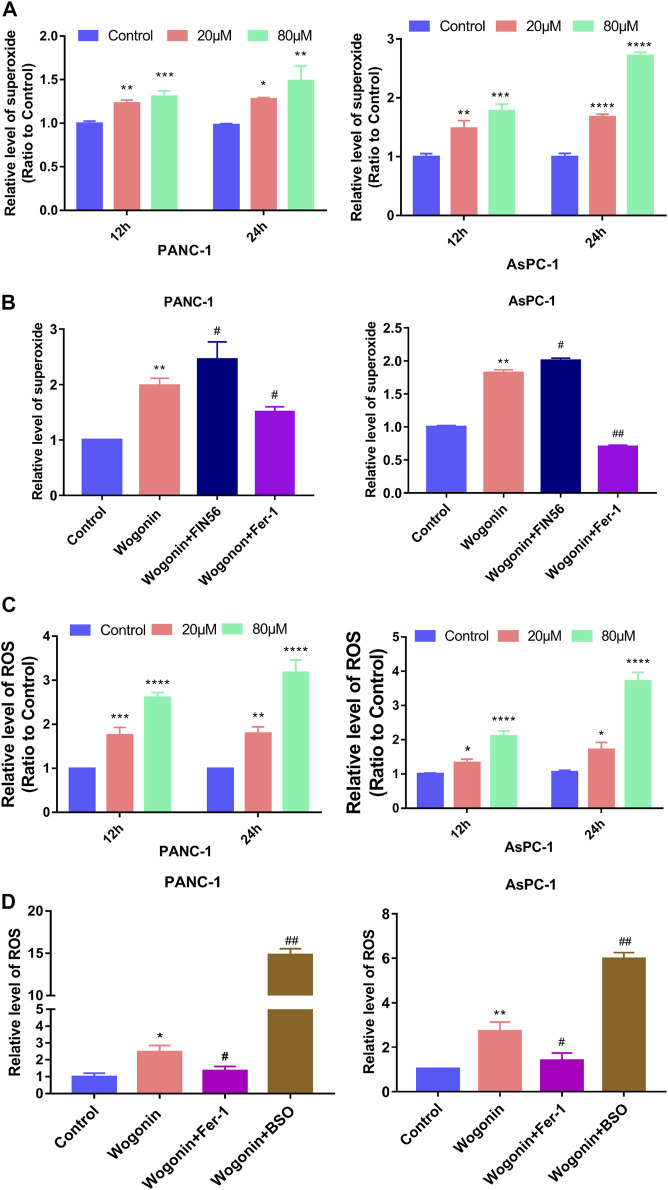
Wogonin-induced accumulation of superoxide and ROS. **(A)** Wogonin induced over generation of superoxide in pancreatic cancer cells in a dosage- and time-dependent manner. **(B)** Wogonin-induced increase of superoxide was mitigated by fer-1, but was reinforced by FIN56. **(C)** Wogonin induced accumulation of ROS in pancreatic cancer cells in a dosage- and time-dependent manner. **(D)** Wogonin-induced accumulation of ROS was alleviated in the presence of Fer-1, but was improved by BSO. *p < 0.05 versus control group; **: *p* < 0.01 versus control group; ***: *p* < 0.001 versus control group; ****: *p* < 0.0001 versus control group; #*p* < 0.05 versus wogonin-alone group; ###*p* < 0.001 versus wogonin-alone group.

Lipid peroxidation can be initiated by the Fenton reaction involving hydrogen peroxide (H_2_O_2_), considering that H_2_O_2_ can be generated from superoxide (Gutteridge, 1984). Hence, we determined the effect of wogonin on superoxide production and ROS levels in cells. Compared to the control cells, the superoxide production and the ROS accumulation in cells increased on treated with 20 μM wogonin for 12 h (Figures 5A, B), which became more obvious when the wogonin dose was increased to 80 μM or the incubation time was increased to 24 h. This indicates that wogonin induced the accumulation of superoxide and ROS in cells in a time and concentration-dependent manner.

To investigate if superoxide and ROS were involved in pancreatic cancer cell death after wogonin treatment, the cells were treated with Fer-1 at 1 μM for 1 h, followed by 80 μM wogonin for 24 h. The results revealed that Fer-1 pre-treatment for 1 h could significantly reduce the lipid peroxidation induced by wogonin (Figure 5C). Similarly, pre-treatment with 200 μM BSO or 150 μM FIN56 for 1 h could promote the cytotoxic effect of wogonin on pancreatic cancer cells (Figure 5D). Together, this indicates that superoxide and ROS are involved in wogonin-induced pancreatic cancer cell death.

### Reduced Nrf2 expression is associated with wogonin-induced ferroptosis

**FIGURE 6:**
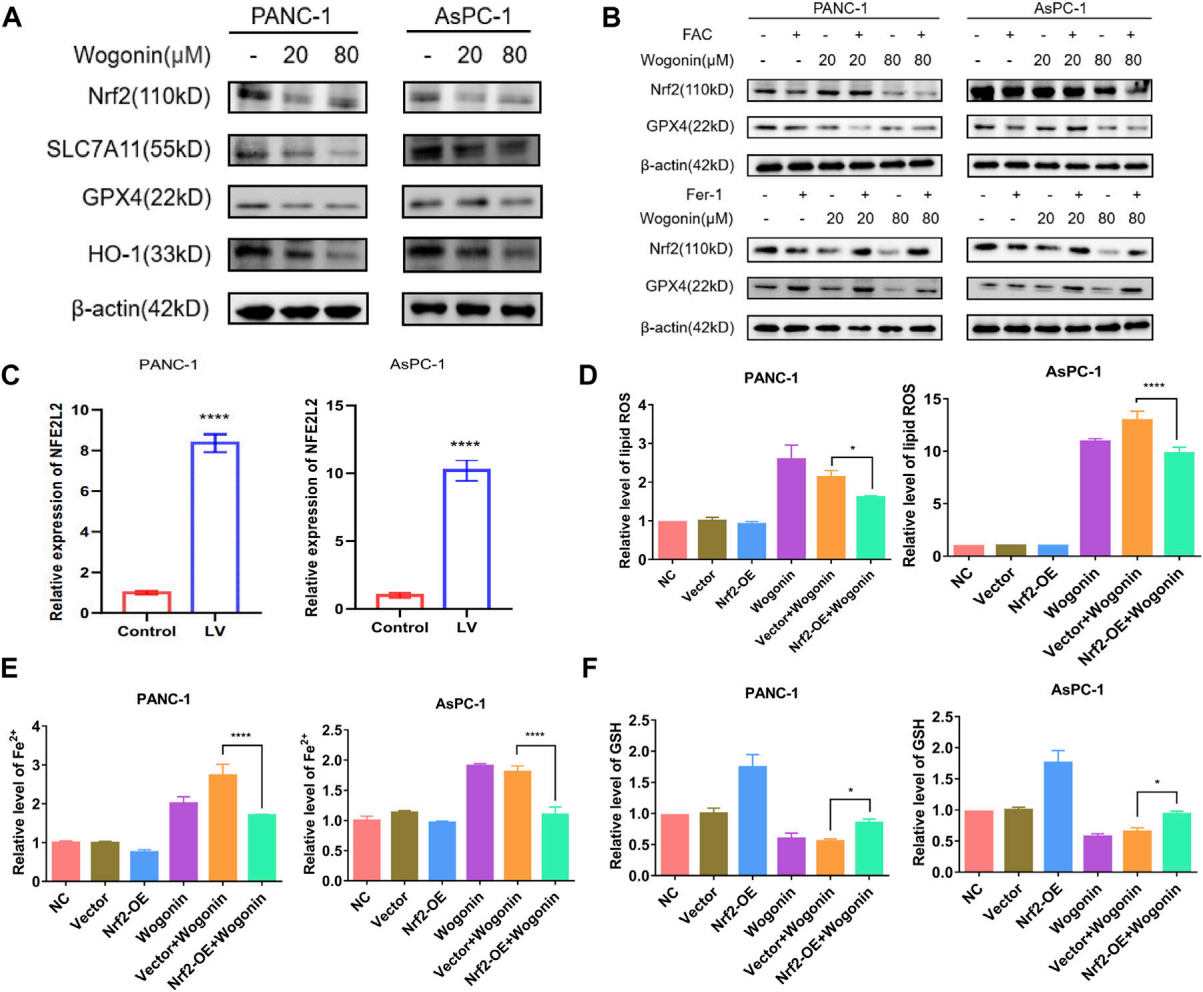
Overexpression of Nrf2 contributed to wogonin-induced ferroptosis in pancreatic cancer cells. **(A)** Western blotting proved that wogonin downregulated the expression of ferroptosis relate protein, in a dosage-dependent manner. **(B)** Effect of ferroptosis inducer or inhibitor on wogonin regulatory protein. **(C)** The overexpression of *NFE2L2* mRNA was detected by RT-qPCR. **(D)** Wogonin-induced accumulation of lipid peroxidation was prevented when Nrf2 was overexpression. **(E)** Overexpression of Nrf2 prevented wogonin-induced overproduction of Fe^2+^. **(F)** Wogonin-induced repression of GSH was improved when Nrf2 was overexpression. *: *p* < 0.05 versus control group; ****: *p* < 0.0001 versus the group treated with wogonin alone.

Nrf2 is a primary regulator of antioxidant responses and plays a vital role in maintaining cellular redox homeostasis. GPX4 and xCT are downstream targets of Nrf2 (Dodson et al., 2019). SLC7A11, also identified as xCT, is the light chain subunit of the cysteine/glutamate reverse transporter system (Koppula et al., 2021). To determine if the Nrf2 signalling pathway is involved in wogonin-induced ferroptosis, we used *NFE2L2*-siRNA to knock down or overexpress the expression of Nrf2, respectively. WB results revealed that wogonin treatment could significantly reduce Nrf2 expression in cells (Figure 6A); however, Fer-1 treatment could reverse this effect (Figure 6B). When *NFE2L2* is overexpressed (Figure 6C), wogonin-induced increase in peroxidation was reversed in cells overexpressing *NFE2L2* (Figure 6D), this inhibitory effect was reversed on knockdown of *NFE2L2* expression in cells (Supplementary Figure S1). Similar results were obtained for the levels of Fe^2+^ in cells, as shown in Figure 6E. Furthermore, *NFE2L2* overexpression increased GSH levels in the cells, which were suppressed following wogonin treatment (Figure 6F). Therefore, these results indicate that wogonin induced ferroptosis by inhibiting *NFE2L2* expression, which induced the accumulation of lipid peroxide and Fe^2+^ in cells and decreased GSH levels.

### Wogonin treatment increased the levels of Fe^2+^ and lipid peroxidation *in vivo*

**FIGURE 7:**
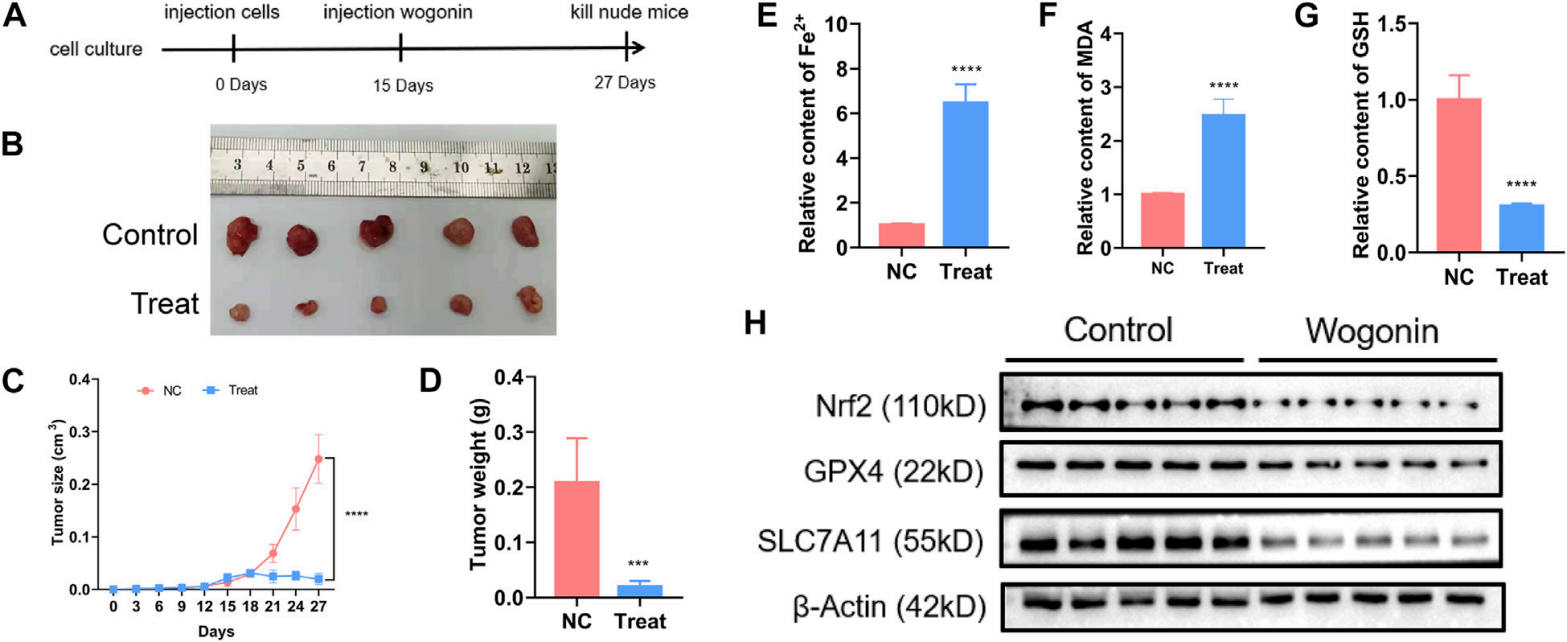
Wogonin improved ferrous iron and lipid oxidation *in vivo*. **(A, B)** Representative images of the nude mice with xenografted pancreatic cancer showed that tumor size was significantly smaller in the mice treated with wogonin at the dosage of 60 mg/kg for consecutive 12 days than that in control group. **(C, D)** Statistical analysis of tumor volumes confirmed as well that wogonin inhibited tumor growth *in vivo*. **(E)** Iron assay showed ferrous iron level was significantly higher in wogonin-treated group than that in control group *in vivo*. **(F)** MDA assay proved that lipid peroxidation became more apparent in wogonin-treated group when compared with control group *in vivo*. **(G)** GSH assay showed GSH level was significantly lower in wogonin-treated group than that in control group *in vivo*. **(H)** Western blotting analysis revealed that wogonin induced marked downregulation of Nrf2, GPX4 and SLC7A11. ***p < 0.001 versus control group; ****p < 0.0001 versus control group.

To verify the lethal effect of wogonin in pancreatic cancer *in vivo*, PANC-1 cells were administered into the left flank of BALB/c nude mice. A reduction in the tumours size was observed in the mice injected with 60 mg/kg of wogonin for 12 days compared to mice in the control group (Figures 7A, B). In the treatment group, the tumour growth was slower, and the tumour weight was lesser compared to the control group (Figures 7C, D). Therefore, wogonin inhibited the growth of tumours *in vivo*. HE staining analysis showed that wogonin had no toxic effect on mouse organs (Supplementary Figure S2). The tumours were harvested on day 12 after the treatment was complete. We analysed whether wogonin treatment could elevate Fe^2+^ ion, malondialdehyde and GSH levels. The results revealed a significant improvement in the levels of Fe^2+^ ion and malondialdehyde in the treatment group compared to the mice in the control group (Figures 7E, F). In the treatment group, a significant reduction in GSH levels was observed compared to the control group (Figure 7G). Similarly, WB results revealed a significant decrease in the expression level of proteins like Nrf2, GPX4, and SLC7A11 in the treatment group (Figure 7H). These results indicate that the wogonin-mediated inhibitory effect on pancreatic cancer cells *in vivo via* ferroptosis.

## Discussion

Pancreatic cancer is a highly malignant gastrointestinal tract tumour. The prognosis of patients with pancreatic cancer is poor, and mortality is high. Targeted and immune therapies in treating pancreatic cancer have improved the patient’s survival rate in the past decade, but the prognosis of these patients continues to remain poor (Bear et al., 2020; Hu et al., 2021). Globally, the incidence and mortality of pancreatic cancer are rising every year, which has become a major public health burden (Ushio et al., 2021).

For the last 2,000 years, *S. baicalensis Georgi* has been part of traditional Chinese medicine (Wang et al., 2018). It has antibacterial, anti-viral, anti-allergic, anti-inflammatory, and anti-tumour properties. Its root contains various bioactive compounds like wogonin, baicalein, baicalin, and oroxylin A (Zhao et al., 2019). Furthermore, nanoparticles and liposomes can improve the bioavailability and stability of wogonin based on its dosage form (Wang et al., 2022). Ferroptosis is an iron-dependent, non-apoptotic type of cell death. Some characteristics of ferroptosis mainly include lipid ROS accumulation and damage caused to cell membranes by oxidative stress, which ultimately leads to cell death (Dixon et al., 2012). Ferroptosis can induce or inhibit tumour cells survival *via* specific drugs by regulating/influencing the expression of associated genes. Studies exploring the role of ferroptosis in cancer treatment are still in the early stages, and its potential clinical applications have become increasingly important. As *KRAS* gene is mutated in about 85%–90% of pancreatic ductal adenocarcinoma (PDAC), it is the main driver of pancreatic cancer (Buscail et al., 2020). “Ferroptosis” was first proposed to describe iron-dependent non-apoptotic cell death in *RAS* mutated cancer cells (Dixon et al., 2012). Theoretically, most PDAC should be sensitive to ferroptosis activators due to the mutant expression of *RAS*-mediated iron metabolism genes, such as *TRFC* (transferrin receptor), *FTH1* (ferritin heavy chain 1) and *FTL1* (ferritin light chain 1) (Yang and Stockwell, 2008). Induction of ferroptosis may be an attractive therapeutic approach for various types of cancer, including pancreatic ductal adenocarcinoma. Therefore, ferroptosis induction in cancer cells is gradually becoming a new therapeutic strategy for pancreatic cancer treatment. Studies have shown that wogonin mediates anti-cancer effects *via* different signalling pathways (Banik et al., 2022); however, its role and effect on the treatment of pancreatic cancer require additional investigation. Moreover, it is still unclear if wogonin mediates its effects *via* the mechanism of ferroptosis in pancreatic cancer cells. Our results show that wogonin can induce ferroptosis in pancreatic cancer cells like PANC-1 and AsPC-1. To the best of our knowledge, our study is the first to explore how wogonin regulates ferroptosis in pancreatic cancers.

Our results revealed that wogonin significantly reduced pancreatic cancer cell survival *in vitro* and *in vivo*, accompanied by an abnormal increase in the levels of Fe^2+^ ions, lipid peroxidation, ROS, and superoxide in cells and a decrease in GSH levels. *In vitro* experiments have shown that the treatment with ferroptosis inducers like FAC, FIN56 or BSO increases wogonin-induced lipid peroxidation and death of pancreatic cancer cells. Furthermore, Fer-1 or DFO treatment inhibits wogonin-induced pancreatic cancer cell death. Similarly, a study has shown that Fer-1 and DFO inhibit the accumulation of ROS induced by scutellarin, thus confirming that the generation of ROS is vital for enhancing ferroptosis (Ito et al., 2005). Our results have proved that wogonin increases the Fe^2+^ ion levels in cells by increasing the expression of TF and its receptors. As a free radical of oxygen molecules, superoxide spontaneously and disproportionately converts into H_2_O_2_, especially under low pH or during the catalysis of superoxide dismutase (Mintz et al., 2020). In cells, H_2_O_2_ participates in the Fenton reaction to induce ferroptosis (Bedard and Krause, 2007). Our results show that the wogonin increases superoxide levels in cells, which can be inhibited upon treatment with Fer-1, indicating that these superoxide levels come from the ferroptosis pathway.

A classical way to induce ferroptosis is to inhibit GSH synthesis and its use (Uberti et al., 2020). Our results revealed a significant reduction in GSH levels in pancreatic cancer cells treated with wogonin in a time- and concertation-dependent manner. Nrf2 is a stress-induced transcription factor, which targets genes that express proteins and enzymes responsible for preventing lipid peroxidation and removing intracellular Fe^2+^. In our study, *NFE2L2* overexpression significantly inhibits lipid peroxidation, Fe^2+^ ion levels, and GSH depletion induced by wogonin in pancreatic cancer cells. Moreover, the knockdown of *NFE2L2* expression using siRNA significantly increases the lipid peroxidation, Fe^2+^ ion levels, and GSH depletion induced by wogonin in pancreatic cancer cells.

**FIGURE 8:**
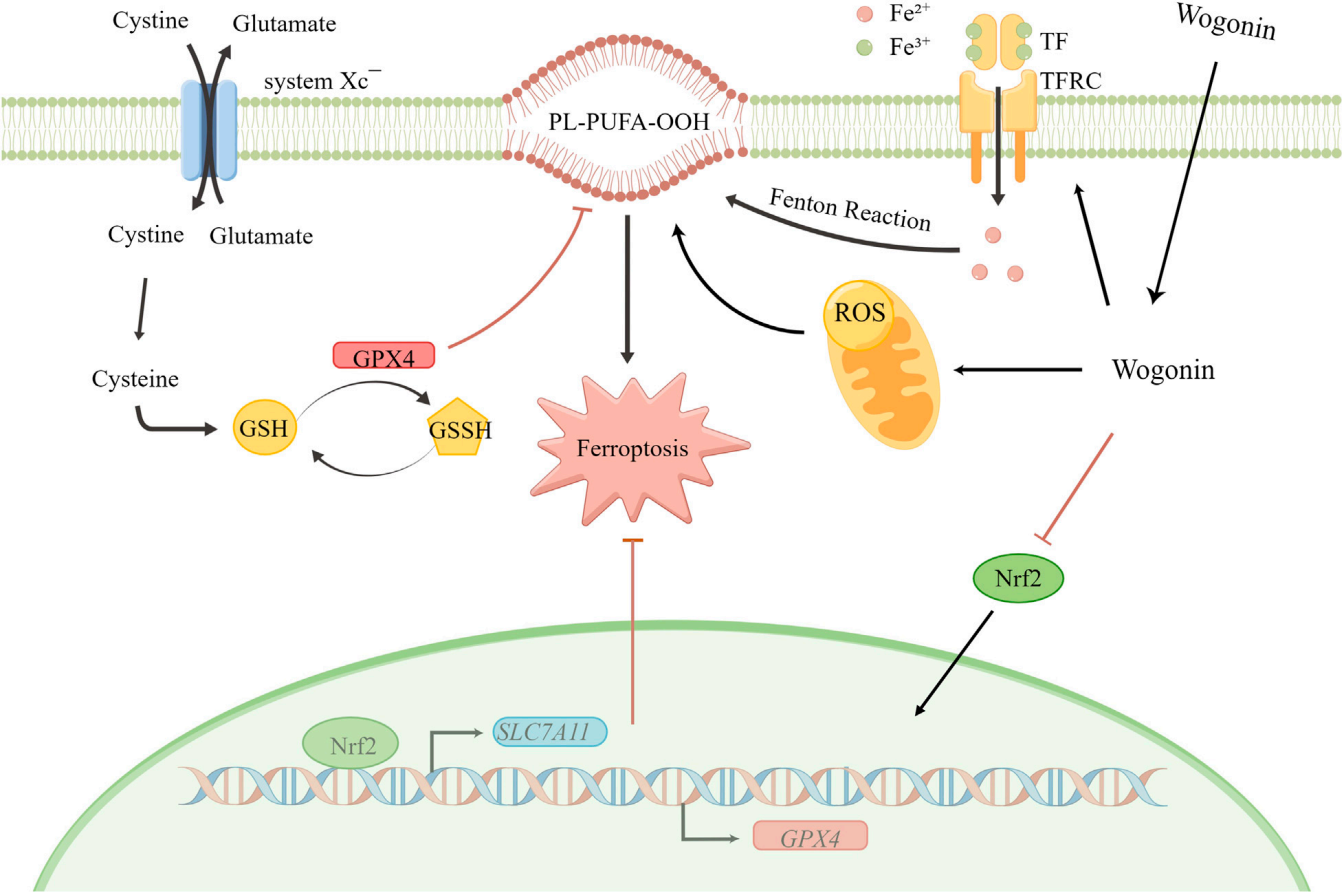
Schematic diagram for wogonin-induced ferroptosis in pancreatic cancer cells.

Furthermore, Nrf2 regulates GSH levels in cells by enhancing *GPX4* and *SLC7A11* expression. Our results show a direct correlation between Nrf2 expression and the sensitivity of cells to ferroptosis since an increase in Nrf2 expression inhibits ferroptosis. Similarly, a study has shown that decreased Nrf2 expression increases the cancer cell sensitivity to ferroptosis inducers (Dodson et al., 2019). Furthermore, inhibiting the Nrf2 signalling pathway could reverse the drug resistance and increase the sensitivity of pancreatic cancer cells to chemotherapy (Zhou et al., 2019; Kim et al., 2020). Therefore, targeting the Nrf2 signalling pathway could be a potential strategy for pancreatic cancer treatment. Our results showed a decrease in the expression of SLC7A11, GPX4, HO-1, and Nrf2 protein on treatment with wogonin in a time and concentration-dependent manner. Furthermore, in pancreatic cancer cells treated with both FAC and wogonin, a significant decrease in Nrf2 and GPX4 protein expression was observed compared to cells treated with FAC alone. Treatment with Fer-1 can reverse Nrf2 and GPX4 expression in cells induced by wogonin. Therefore, wogonin induces lipid peroxidation by activating *TF* and *TFRC* in an iron-dependent manner but also decreases GSH levels in cells by decreases GSH levels in cells *via* Nrf2-GPX4 pathway, thereby inducing ferroptosis in pancreatic cancer cells (Figure 8). Tumor is a threat to human life and health, currently commonly used chemotherapy drugs to tumor patients, kill a large number of malignant cells at the same time, often bring a fatal blow to the body’s immune function. Therefore, the search for natural drugs with high efficiency and low toxicity that not only have anti-tumor activity but also can enhance immune function will eventually bring new hope to patients with malignant tumors. The above research results show that the crude drug element of tumor cell killing effect has a unique mechanism of action, and also have the function of the efficient and low toxicity, so the prevention of tumor, and tumor drug treatment with combination chemotherapy alone has a broad application prospect. However, there are still some limitations in this study. This study did not set up a positive control group of ferroptosis inducer alone, only studied the effect of wogonin combined with positive control on ferroptosis, and did not detect sham drugs after Nrf2 knockdown or overexpression in animals. These need to be follow-up for a deeper research.

## Conclusion

Wogonin induces pancreatic cancer cell death *via* ferroptosis. Wogonin can increase Fe^2+^ ion levels in cells by increasing TF levels. The increase in Fe^2+^ ion levels in cells enhances the Fenton reaction, which increases the production of ROS and increases lipid peroxidation. In addition, wogonin decreases GSH levels in cells *via* the Nrf2-mediated GPX4 pathway, thereby further enhancing lipid peroxidation and ROS accumulation in pancreatic cancer cells.

## Data Availability

The original contributions presented in the study are included in the article/Supplementary Material, further inquiries can be directed to the corresponding author.

## Supplementary material

SUPPLEMENTARY FIGURE S1 Knockdown of Nrf2 contributed to wogonin-induced ferroptosis in pancreatic cancer cells. **(A)** RT-qPCR analysis was performed to detect *NFE2L2* mRNA expression in PAAD cells transfected with or without si-*NFE2L2*. **(B)** Wogonin-induced accumulation of lipid peroxidation was prevented when Nrf2 was knockdown. **(C)** Knockdown of Nrf2 prevented wogonin-induced overproduction of Fe^2+^. **(D)** Wogonin-induced repression of GSH was improved when Nrf2 was knockdown. *: *p* < 0.05 versus control group; ****: *p* < 0.0001 versus the group treated with wogonin alone.

SUPPLEMENTARY FIGURE S2 H&E staining for orthotopic xenograft tumor sections (× 200).
