# Supplementary figures and images for: Wogonin induces ferroptosis in pancreatic cancer cells by inhibiting the Nrf2/GPX4 axis
Source: Front Pharmacol. 2023 Feb 22;14:1129662. doi: 10.3389/fphar.2023.1129662 (PMC9992170; doi:10.3389/fphar.2023.1129662)

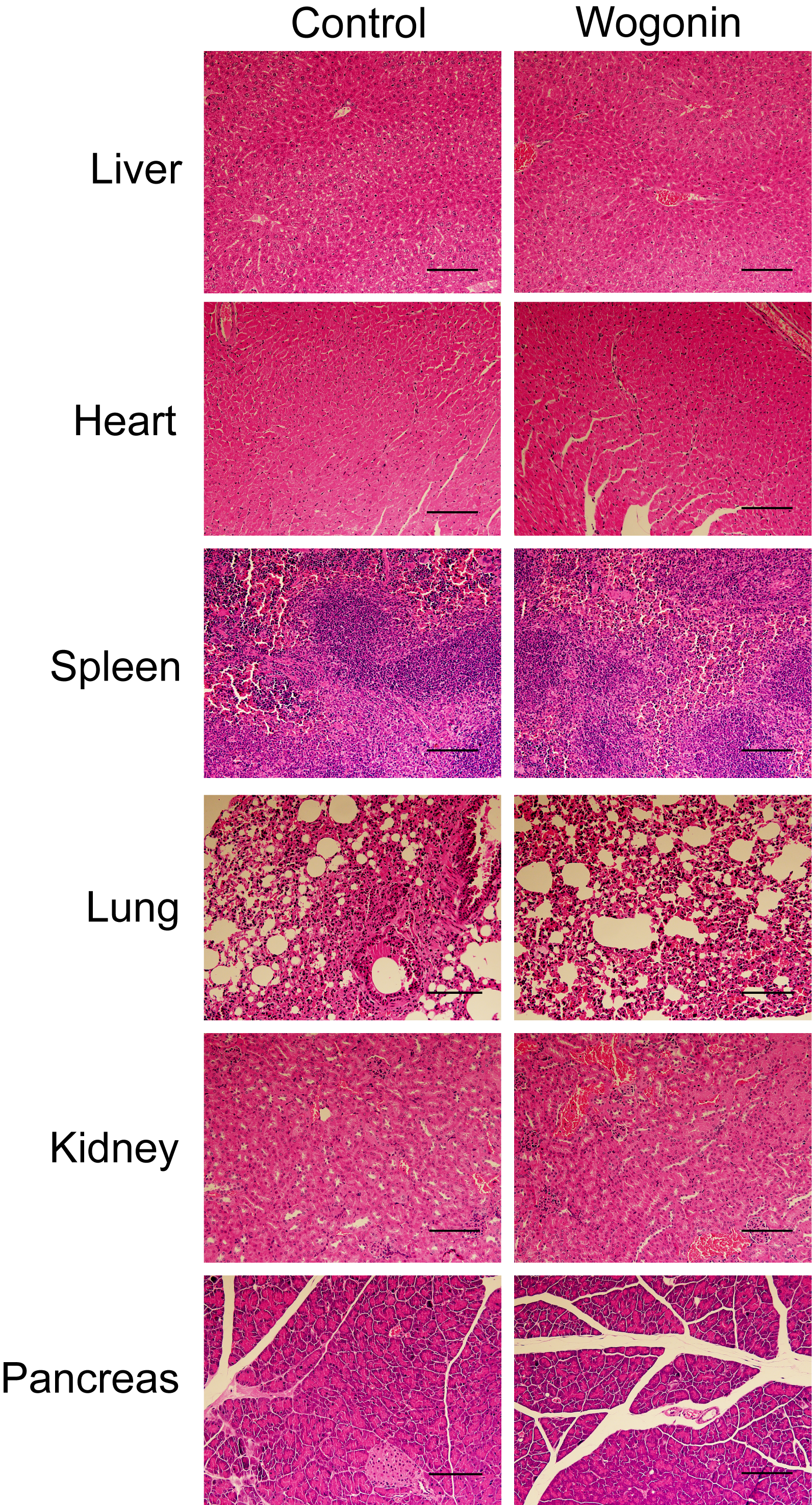

Supplement: Supplementary file 1 [file Image2.TIF]

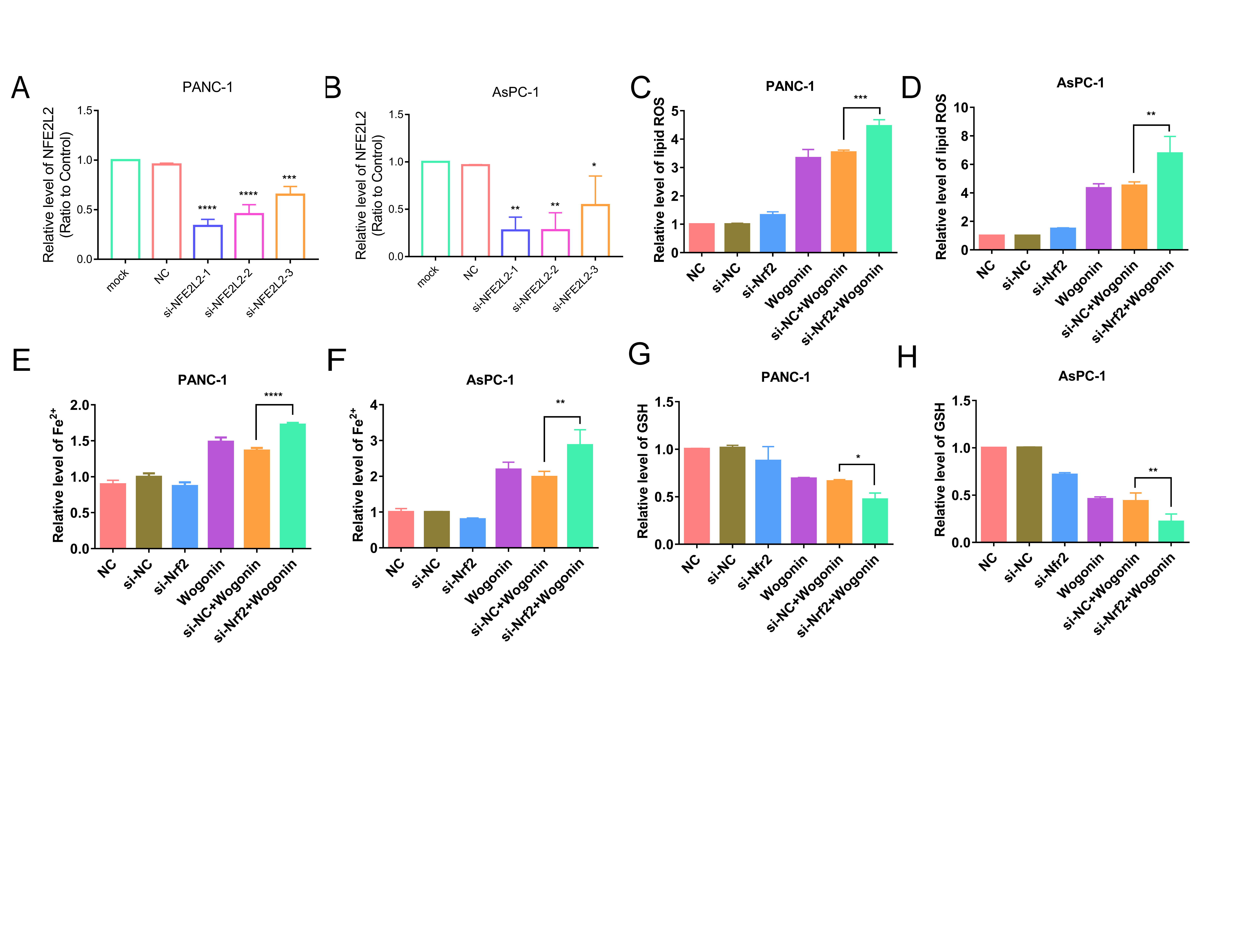

Supplement: Supplementary file 2 [file Image1.TIF]
